# Protective effects of citrulline supplementation in ulcerative colitis rats

**DOI:** 10.1371/journal.pone.0240883

**Published:** 2020-10-16

**Authors:** Bin Cai, Min-hong Zhou, Hua-li Huang, A-cheng Zhou, Zheng-da Chu, Xiao-dong Huang, Chun-wei Li

**Affiliations:** 1 Department of Anorectal Surgery, Wuxi Traditional Chinese Medicine Hospital, Wuxi, Jiangsu, People’s Republic of China; 2 Department of Gastroenterology, Wuxi Traditional Chinese Medicine Hospital, Wuxi, Jiangsu, People’s Republic of China; Icahn School of Medicine at Mount Sinai, UNITED STATES

## Abstract

It has been reported that supplementing certain amino acids has therapeutic effects on ulcerative colitis (UC). We intend to explore whether citrulline (Cit) supplementation has protective effects on UC. Fifteen male Wistar rats were divided into normal control group (NC group), UC group and UC+Cit group, with five rats in each group. The UC model was established by TNBS/ethanol method. Rats in UC+Cit group were intragastrically administered with Cit for 7 consecutive days after modeling. All rats were sacrificed after 7 days. Blood samples were collected to detect the number of monocytes. Colon tissues were taken for HE staining. Immunohistochemistry staining for CD68 and p-STAT3 were performed to detect the infiltration of monocytes and the phosphorylation of STAT3 in colon tissues. The concentrations of MCP-1, IL-6 and IL-17A and the protein expression of p-STAT3 in colon tissues were measured by ELISA and western blot methods, respectively. The body weight of UC group rats decreased significantly after 7 days (*p*<0.05). However, the weight loss of UC+Cit group rats was not statistically significant (*p*>0.05). The number of peripheral blood monocytes in UC+Cit group was significantly lower than that in UC group (*p*<0.05), and the infiltration of CD68-positive monocytes in the colon tissue of UC+Cit group was significantly reduced than that in UC group. The concentrations of MCP-1, IL-6 and IL-17A and the expression of p-STAT3 in colon tissues of UC+Cit group rats were significantly lower than those in UC group (both *p*<0.05). Our study suggests that Cit supplementation may be a potential therapy for UC.

## Introduction

Ulcerative colitis (UC) is a chronic non-specific inflammatory disease of the intestine but the cause is not fully elucidated. The lesion mainly occurs in the rectum and sigmoid colon, even retrogrades to the proximal end of the colon. It is reported that the incidence of UC in North America and Europe was 23.14/100,000 and 57.9/100,000, respectively, and the prevalence of UC was up to 505/100,000 in Europe (Norway) and 319/100,000 in North America (Canada) [1]. The pathogenesis of UC is complex, which involves genetics, immunity, infection, and environmental factors. The clinical treatment of UC mainly includes aminosalicylic drugs, adrenal glucocorticoids and immunosuppressants, but these drugs may have certain adverse reactions.

Therefore, some researchers turn their attention to the potential therapeutic effect of amino acids supplementation on UC. Coburn et al. [2] reported an abnormal utilization and metabolism of arginine (Arg) in the process of UC, and the level of Arg in the intestinal tissue of patients with active UC was significantly reduced. Arg supplementation improved the clinical parameters of survival, body weight loss, and colon weight loss, and reduced the expression of pro-inflammatory cytokines and chemokines. Meanwhile, Arg treatment also increased the migration ability of colon epithelial cells and improved the repair ability of intestinal mucosa in dioctyl sodium sulfosuccinate (DSS) induced UC mice [3]. Arg is a kind of non-essential amino acid, and its sources include absorption in digestive tract, production by protein decomposition, and endogenous synthesis through amino acid metabolism. It is noted that Arg undergoes first-pass metabolism via the gastrointestinal tract and liver. Meanwhile, the inflammatory state of the UC intestine also affects the absorption of Arg, so the bioavailability and conversion efficiency of Arg supplementation in UC patients is relatively low.

Citrulline (Cit) is a non-protein alpha-amino acid, which acts as the precursor amino acid of Arg. It can catalyze the synthesis of Arg by arginosuccinate synthetase (ASS) and argininosuccinate lyase (ASL). Moreover, Cit has the advantage of not being decomposed by the liver, so its bioavailability is higher than Arg. A recent study confirmed that Cit supplementation was more efficient at increasing Arg availability than was Arg supplementation itself in normal mice [4]. Therefore, we intended to explore whether Cit supplementation has a protective effect on UC.

## Materials and methods

### Animals

Fifteen male Wistar rats, weighing 220±20 g, were purchased from Cavans Experimental Animal Company (Changzhou, China). All rats were housed in the experimental animal center of Wuxi Institute of Translational Medicine. The house temperature was kept at 23–25°C, humidity at 45%-55%, with a 12-hour light/12-hour dark light cycle. All experimental procedures were approved by the Ethics committee of Wuxi Traditional Chinese Medicine Hospital.

### UC model

After 7 days of adaptive feeding, the UC model was established by 2,4,6-trinitrobenzenesulfonic acid (TNBS)/ethanol method according to the reported literature [5]. The specific method was as follows: An equal volume of 5% TNBS and 50% ethanol were mixed as the molding agent. The rats were weighed and anesthetized with isoflurane inhalation. After anesthesia, an 8 cm rubber hose was inserted from the anus into the intestine. Through the rubber hose, the TNBS/ethanol modeling agent was slowly injected into the intestinal cavity of rats at the dose of 4 mL•kg^-1^. After the injection, lift the tail of the rat and invert it for 30 seconds. The rats were put back into the cage and given free diet after waking up. The general condition and survival status of rats were observed every 8 hours after modeling.

### Study design

Rats were randomly divided into 3 groups, namely normal control group (NC group), UC group and UC+Cit group, with 5 rats in each group. Rats in UC group and UC+Cit group were modeled according to the above modeling method. From the first day after modeling, NC group and UC group rats were intragastrically administered with 4.0 mL saline for 7 consecutive days, and UC+Cit group rats were intragastrically administered with Cit (900 mg•kg^-1^, at a volume of 4 mL).

### General condition observation and weight measurement

The general condition of the rats was observed every day, including: mental state, activity, drinking water consumption, stool characteristics, stool color, blood in the stool. Weight change of the rats was measured every four hours. When the body weight of rats was reduced by more than 25% compared with the initial weight, the mental state of rats was extremely poor, and the rats had difficulty in feeding and drinking by themselves, we would give them euthanasia treatment.

### Sample collection

After 7 consecutive days of intragastric administration, the rats were fasted for 12 hours. After isoflurane anesthesia, blood sample was obtained by cardiac puncture. Rats were sacrificed by spinal dislocation, and then colon tissues were collected. Part of the harvested colon tissue was immediately frozen at -80°C for further testing, and the remaining part was fixed with 4% paraformaldehyde solution, stained with hematoxylin-eosin (HE) for histopathology observation.

### Assessment of histopathological score (HS)

The histopathological score of colon injury was performed according to the criteria reported previously by Cooper et al [6]. HS included the following criteria: crypt damage (0 = none; 1 = basal 1/3 damage; 2 = basal 2/3 damage; 3 = crypt lost, surface epithelium present; 4 = crypt and surface epithelium lost), Tissue inflammation (0 = no visible enlargement of lamina propria; 1 = mild enlargement of lamina propria; 2 = moderate expansion of lamina propria with increased infiltration of inflammatory cells; 3 = substantial increase in lamina propria size in addition to heavy infiltration of nuclei into surrounding areas). The histopathological score was assessed by two independent observers from the department of pathology who were blind to the purpose of our study.

### Detection of the number of monocytes and lymphocytes

Rat blood collected in EDTA anticoagulant tube was used to detect the number of monocytes and lymphocytes by Coulter LH 750 automatic blood cell analyzer (Beckman Coulter, USA).

### Enzyme-Linked Immunosorbent Assay (ELISA)

The frozen colonic tissues were homogenized and lysed in cell lysis buffer (Cell Signaling Technology, USA), and centrifuged at 14000 g, at 4°C for 10 min. The supernatant was then collected. The concentrations of monocyte chemoattractant protein-1 (MCP-1), interleukin (IL)-6 and IL-17A in the colon tissues were detected via the corresponding Rat MCP-1, IL-6 and IL-17A ELISA Kits (4A Biotech, Beijing, China) according to the manufacturer’s protocols. The absorbance values at 450 nm were measured using microplate reader (Biotek, USA).

### Immunohistochemistry

Colon tissues were fixed with 4% paraformaldehyde for 48 hours, embedded in paraffin, and sliced at a thickness of 4 μm. After dewaxing, the slices were incubated with 3% hydrogen peroxide to inactivate endogenous peroxidase and sealed with 5% BSA (Gibco, USA) for 1 hour. The slices were incubated overnight at 4°C with the primary antibodies (CD68, 1:100, Abcam, UK; p-STAT3, 1:50, Abcam, UK), and then incubated at 37°C with the Goat Anti-Rabbit IgG second antibody (1:100, Abcam, UK). The color reaction was developed using 3,3′–diaminobenzidine (DAB) solution and counterstained with hematoxylin. The slices were observed with an Olympus IX51 microscope (Olympus, Japan) and analyzed with Image J software (National Institutes of Health, Bethesda, MD, USA). The positive staining area (%) of the colon tissue in each group was calculated.

### Western blot

The fresh frozen rat colon tissue was homogenized in RIPA buffer (Beyotime Biotechnology, China) with 1% protease inhibitor (Roche, Switzerland) and phosphatase inhibitor (Roche, Switzerland). Centrifuge the homogenate for 30 min at 4°C at 12,000 g, and then collect the protein supernatant. Protein concentration was measured by BCA Protein Assay Kit (Pierce, USA). Protein samples were mixed with 5×Loading buffer, boiled for 10 min at 95–100°C, and then stored at -20°C for further detection. A total of 100 μg of protein was loaded into 8% SDS-PAGE (Beyotime Biotechnology, China) and transferred onto PVDF membranes (Millipore, UK). After blocked with 5% skimmed milk for 1 hour, the membranes were incubated overnight with primary antibodies against p-STAT3 (1:2000, Abcam, UK), STAT3 (1:2000, Abcam, UK) and β-actin (1:5000, Abcam, UK) in a shaking table at 4°C. After washing three times with TBST, the membranes were incubated with the Goat Anti-Rabbit IgG secondary antibody (1:2000, Abcam, UK) for 1 hour at room temperature. Protein bands were visualized by the ChemiScope 3500 Mini Imaging System (Clinx Science Instruments, China).

### Statistics

All data was expressed as means ± SEM. Statistical analyses were performed with GraphPad Prism 5.0 (San Diego, CA). Comparisons between multiple groups were made with one-way ANOVA. When comparisons between two independent samples were made, Student’s t test was performed. *P*< 0.05 was considered statistically significant.

## Results

### Protective effects of citrulline supplementation on weight loss and colonic histopathological damage in UC rats

**Fig 1:**
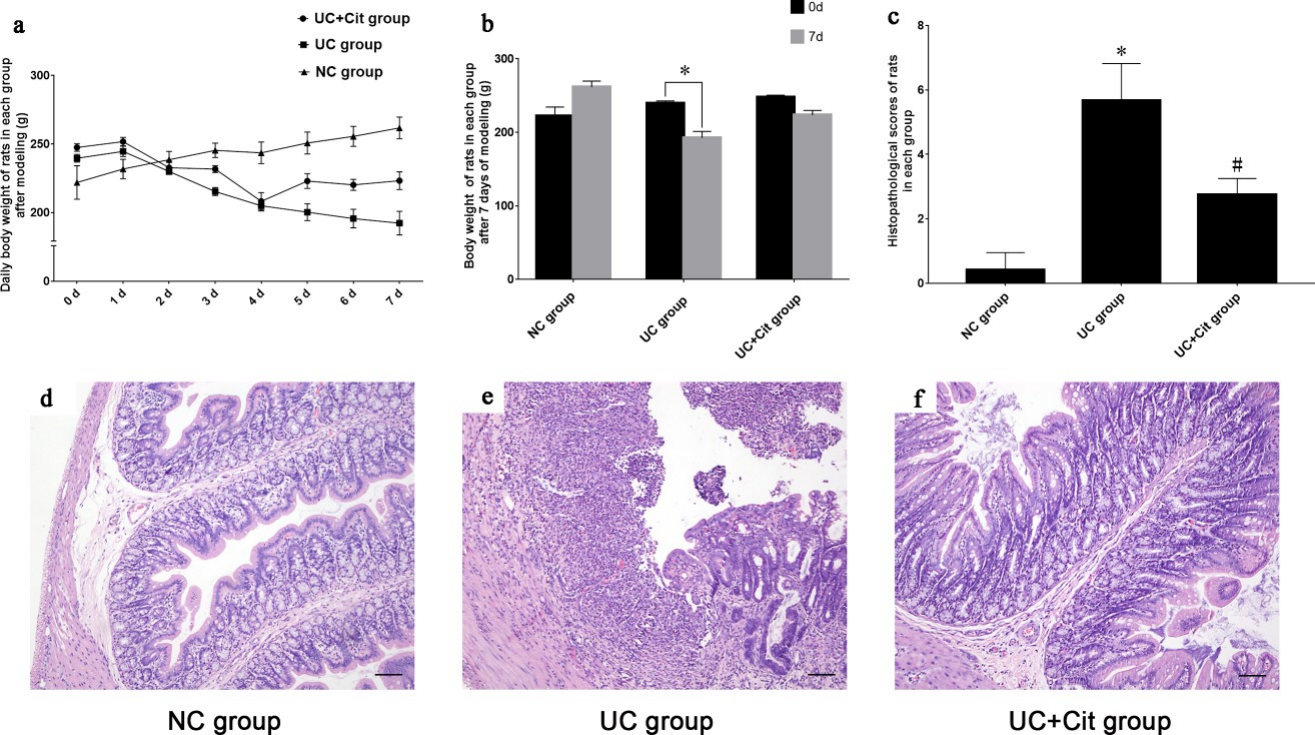
Body weight changes of rats and histopathology changes of colon tissues in each group. Daily body weight of rats in NC group, UC group and UC+Cit group from 0th to 7th day after modeling (a). Body weight of UC group rats significantly decreased on the 7th day after modeling, *P<0.05 *vs*. the initial body weight at day 0. Weight of rats in UC+Cit group also decreased on the 7th day after modeling, but there was no significant difference from the initial weight (b). The histopathology score of UC group rats was significantly higher than that of NC group (*P<0.05), while the score of UC+Cit group was significantly lower than that of UC group (#*P*<0.05) (c). In the NC group, the epithelial cells were neatly arranged, goblet cells were abundant, gland structure was clear, capillaries and lymphocytes were scarce (d) (×100). Ulcerative defects were observed in the mucosal epithelium of the UC group, with mucosal hyperemia, obvious destruction of glandular structure, significant reduction of goblet cells, and a large number of inflammatory cell infiltration (e) (×100). The intergrity of mucosal epithelium cells, glandular structure and goblet cells were retained in UC+Cit group rats, with slight hyperemia and fewer infiltrated inflammatory cells around (f) (×100).

After 7 consecutive days of intragastric administration, 5 rats in the NC group, 3 rats in the UC group and 4 rats in the UC+Cit group survived, respectively. S1 Fig showed the survival curve of each group. The body weight of NC group rats increased gradually through the 7 days; while the weight of UC and UC+Cit group rats began to decrease 2 days after modeling (Fig 1A). Compared with the initial body weight at day 0, the body weight of UC group rats was significantly decreased on the 7th day after modeling (*P*<0.05). However, there was no statistically significant difference in the weight of UC+Cit group rats between day 0 and day 7 (*P*>0.05), suggesting that Cit supplementation could partially alleviate the weight loss of UC rats (Fig 1B). Histopathological score assessment results showed that the score of colon injury in UC group rats was significantly higher than that in NC group (*P*<0.05), while the score of colon injury in UC+Cit group was significantly lower than that in UC group (*P*<0.05) (Fig 1C). Cit supplementation could reduce the inflammatory damage of colon tissue in UC rats. Histopathology changes of colon tissues of rats in each group were showed in Fig 1D–1F.

### Citrulline supplementation reduced the number of monocytes in peripheral blood and inhibited the infiltration of monocytes in the colon tissue of UC rats

**Fig 2:**
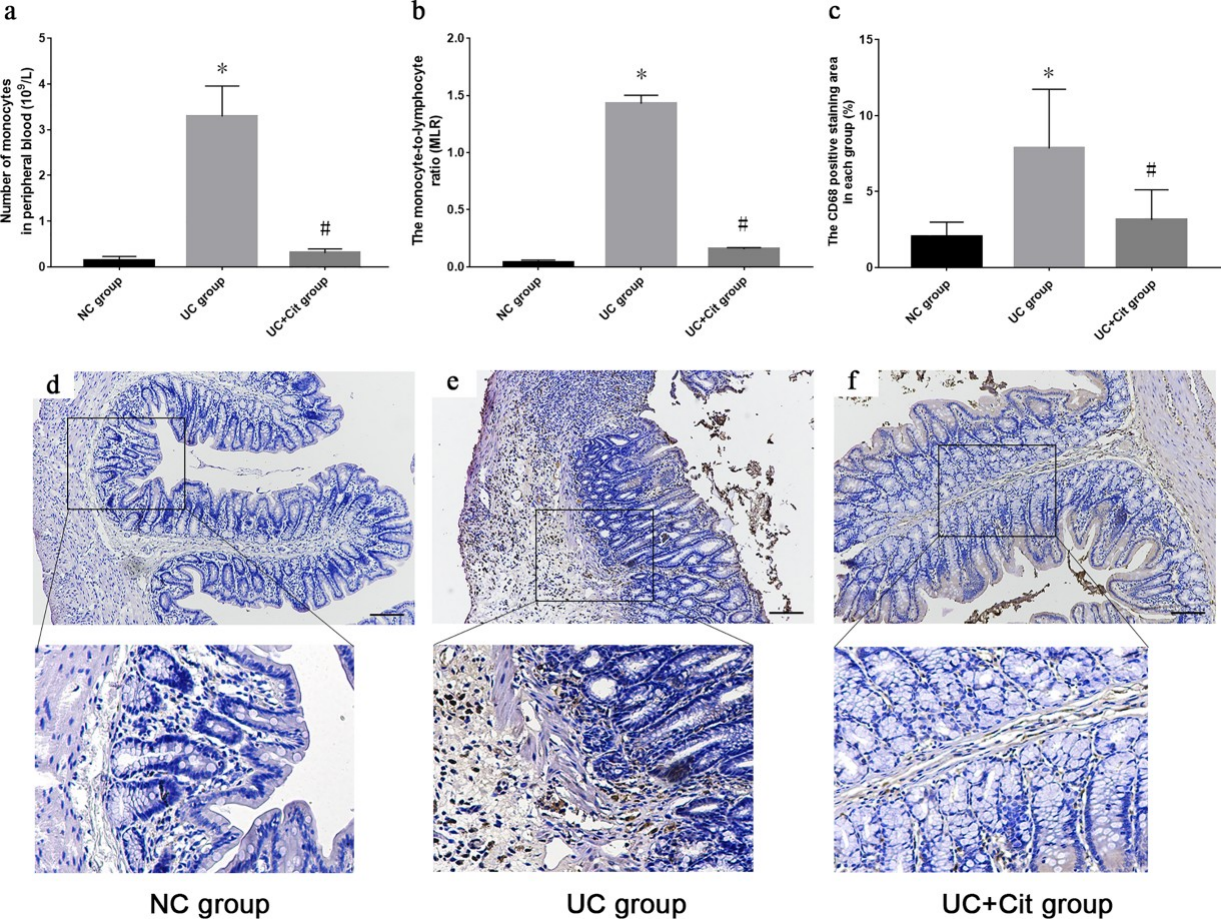
Comparison of the number of monocytes in peripheral blood and the infiltration of monocytes in colon tissues of each group. The number of monocytes in peripheral blood of UC group was significantly higher than NC group (*P<0.05 *vs*. NC group). The number of monocytes in peripheral blood of UC+Cit group was significantly lower than UC group (#*P*<0.05 *vs*. UC group) (a). The monocyte-to-lymphocyte ratio (MLR) of UC group was significantly higher than that of NC group (*P<0.05 *vs*. NC group), and the MLR of UC+Cit group was significantly lower than that of UC group (#*P*<0.05 *vs*. UC group) (b). The CD68 positive staining area (%) in UC group was significantly larger than that in NC group (*P<0.05 *vs*. NC group), and the CD68 positive staining area (%) in UC+Cit group was significantly smaller than that in UC group (#*P*<0.05 *vs*. UC group) (c). Infiltration of CD68-positive monocytes in colon tissues of rats in NC groups (d), UC group (e) and UC+Cit group (f) (×100).

Compared with NC group, the number of monocytes in peripheral blood of UC group rats was significantly increased (*P*<0.05). The number of monocytes in peripheral blood of UC+Cit group rats was significantly lower than that of UC group (*P*<0.05) (Fig 2A). In addition, the monocyte-to-lymphocyte ratio (MLR) of UC group was significantly higher than that of NC group (*P*<0.05), while the MLR of UC+Cit group was significantly lower than that of UC group (*P*<0.05) (Fig 2B). The CD68 positive staining area (%) in the colon tissue of UC group was significantly increased than that of NC group (*P*<0.05), and the CD68 positive staining area (%) in the colon tissue of UC+Cit group was significantly less than that of UC group (*P*<0.05) (Fig 2C). Immunohistochemically staining for CD68 showed an increase of CD68-positive monocytes in the colon tissue of UC group rats compared with NC group rats; however, such infiltration was not observed in the Cit supplemented rats (Fig 2D–2F).

### Citrulline decreased the concentrations of MCP-1, IL-6 and IL-17A in the colon tissue of UC group rats

**Fig 3:**
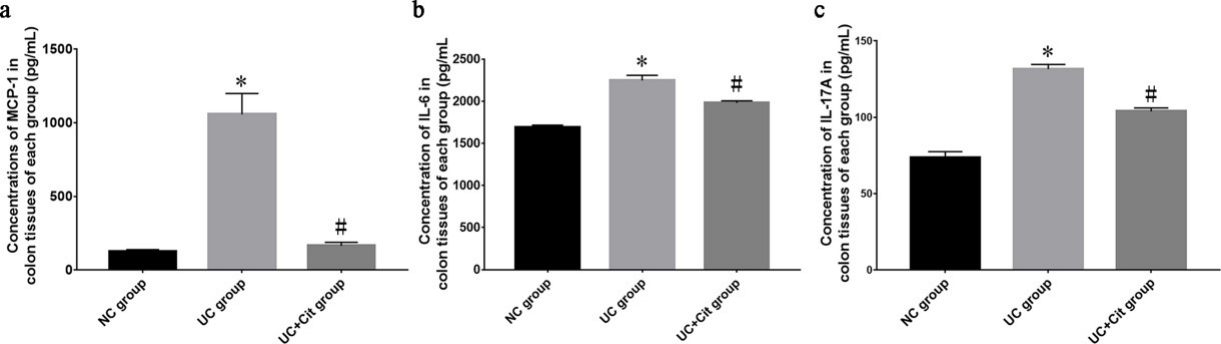
Comparison of MCP-1, IL-6 and IL-17A concentrations in colon tissues of each group. The concentrations of MCP-1 (a), IL-6 (b) and IL-17A (c) in colon tissues of UC group were significantly higher than those of NC group (*P<0.05 *vs*. NC group). The concentrations of MCP-1 (a), IL-6 (b) and IL-17A (c) in colon tissues of UC+Cit group were significantly lower than those of UC group (#*P*<0.05 *vs*. UC group).

ELISA analysis showed that the concentrations of MCP-1, IL-6 and IL-17A in the colon tissue of UC group rats were significantly higher than those of NC group rats (*P*<0.05). However, the concentrations of MCP-1, IL-6 and IL-17A in the colon tissue of UC+Cit group rats were significantly lower than those of UC group rats (all *P*<0.05) (Fig 3A–3C).

### Citrulline supplementation inhibited the phosphorylation of STAT3 in the colon tissue of UC rats

**Fig 4:**
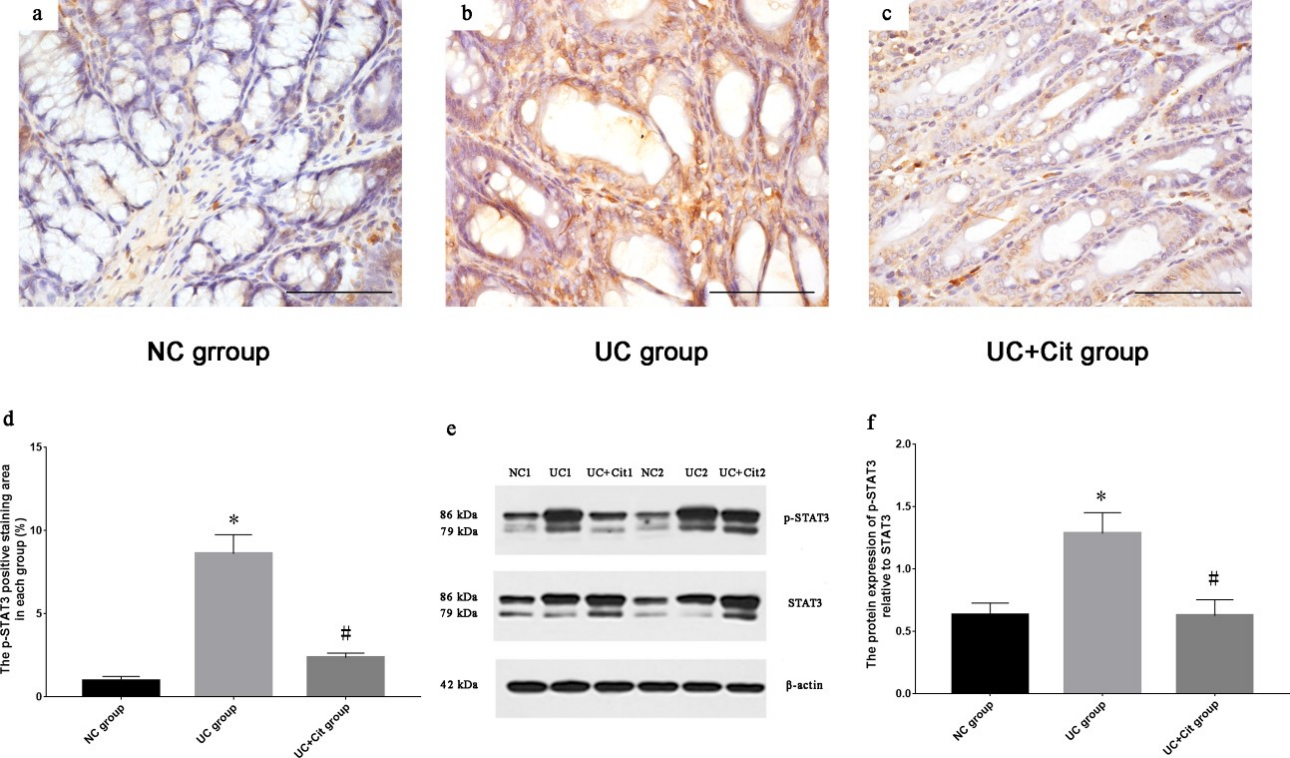
Comparison of phosphorylation of STAT3 in colon tissues of each group. Immunohistochemically staining for p-STAT3 in colon tissues of NC group (a), UC group (b) and UC+Cit group (c) (×400). The p-STAT3 positive staining area (%) in UC group was significantly larger than that in NC group (*P<0.05 *vs*. NC group), and the p-STAT3 positive staining area (%) in UC+Cit group was significantly smaller than that in UC group (#*P*<0.05 *vs*. UC group) (d). The expression of p-STAT3 protein relative to STAT3 protein was determined by western blot analysis (e). Expression of p-STAT3 protein in colon tissues of UC group was significantly higher than that of NC group (*P<0.05 *vs*. NC group), and expression of p-STAT3 protein in UC+Cit group was significantly lower than that of UC group (#*P*<0.05 *vs*. UC group) (f).

MCP-1 is a downstream target of the signal transducer and activator of transcription 3 (STAT3) signaling pathway. The production of MCP-1 could be reduced by inhibiting the phosphorylation of STAT3 [7]. Immunohistochemical analysis of the phosphorylation of STAT3 in the colon tissue of UC rats showed that the p-STAT3 positive staining area (%) in UC group was significantly larger than that in NC group (*P*<0.05), and the p-STAT3 positive staining area (%) in UC+Cit group was significantly smaller than that in UC group (*P*<0.05) (Fig 4A–4D). Western blot analysis showed that p-STAT3 protein expression in colon tissues of UC group was significantly higher than that of NC group (*P*<0.05). And compared to UC group, UC+Cit group colon tissues showed significant lower p-STAT3 protein expression levels (*P*<0.05) (Fig 4E and 4F).

## Discussion

Amino acids are important nutrients in vivo, which can be utilized to produce proteins and various metabolites, such as nitric oxide, polyamines, and other molecules. Amino acids are essential for intestinal growth, maintenance of mucosal integrity and barrier function. Moreover, amino acids are energy substrates for intestinal cells and the material basis for wound healing of intestinal mucosa [8]. Several studies have demonstrated that there were abnormalities in amino acids production and metabolism in UC, including glutamine (Gln), glutamate (Glu), tryptophan (Trp), etc. Supplementing amino acids had a protective effect on UC, and its mechanism involved in reducing inflammation, inhibiting oxidative stress and reducing the expression of pro-inflammatory cytokines [2, 9, 10]. A growing number of researchers have suggested that restoring amino acids supply and metabolism may be a promising adjuvant therapy for UC.

It has been demonstrated that the level of Arg was decreased in the colon tissue of UC patients and negatively correlated with the disease activity [2]. Arg supplementation could improve the clinical parameters of survival, body weight loss, and colon weight loss in UC. In addition, Arg treatment could reduce the expression of pro-inflammatory cytokines and chemokines, increased the migration ability of epithelial cells and improved the repair ability of intestinal mucosa [3]. As the precursor amino acid of Arg, Cit supplementation could increase the level of Arg in the body [11]. Cit is a kind of non-protein amino acid which is synthesized mainly by small intestinal epithelial cells in humans. The main physiological function of Cit is its conversion to Arg [12]. In our study the body weight of UC group rats decreased significantly after modeling, while the weight loss of UC rats receiving Cit supplementation was improved after treatment. This result indicated that Cit supplementation could alleviate the weight loss of UC rats. The involved mechanism may be that Cit improved the structural integrity of the intestinal mucosa, thus protecting the absorption function of the intestine. In addition, the histopathology score of UC+Cit group was significantly lower than that of UC group and the concentrations of pro-inflammatory cytokines IL-6 and IL-17A in colon tissues of UC+Cit group were significantly lower than those of UC group. These results suggested that Cit supplementation had a protective effect on colonic inflammatory damage in UC rats.

Monocytes are a subtype of white blood cells that originate from the bone marrow and participate in inflammatory responses by releasing pro-inflammatory cytokines, chemokines and other active mediators. Monocytes can differentiate into macrophages and dendritic cells in tissues and play an important role in innate immunity. Persistent activation of monocytes is involved in the development of UC [13]. Cherfane et al. [14] reported that peripheral blood monocyte count in patients with UC was closely related to the disease activity. Moreover, the researchers proposed that monocyte count could serve as a potential biomarker of UC. Our study found that the count and proportion of peripheral blood monocytes in UC rats supplemented with Cit were significantly lower than those in UC rats without Cit supplementation. In addition, Cit supplementation could reduce the infiltration of monocytes in the colon tissue of UC rats. These results suggested that Cit supplementation may have a protective effect on UC by inhibiting the activation of monocytes.

MCP-1 is a CC chemokine, which plays an important role in recruiting monocytes and macrophages from peripheral blood into inflamed tissues. In addition, MCP-1 also has chemotactic effects on lymphocytes and basophils [15]. A number of studies have shown that the production of MCP-1 was up-regulated in the mucosal tissue of UC patients and UC experimental models, suggesting that MCP-1 played an important role in the infiltration and activation of inflammatory cells in inflamed tissues [16–18]. Khan et al. [15] reported that the severity of colitis and the mortality were significantly reduced in MCP-1 deficient mice, compared with wild-type control mice. In addition, the myeloperoxidase activity, the expression level of IL-1β, IL-12p40 and IFN-γ, as well as the infiltration of CD3+T cells and macrophages in the colonic mucosa were decreased in MCP-1 deficient mice. Meanwhile, a decrease of MCP-1 expression was often reported in studies about the treatment of UC [19, 20]. Our study found a significant decrease in the expression of MCP-1 in the colon tissue of UC rats supplemented with Cit. We suggest that Cit supplementation may reduce the infiltration of monocytes by inhibiting the expression of MCP-1, thereby attenuating the colonic inflammation in UC.

STAT3 is a member of the STAT protein family, which can be phosphorylated by receptor-associated Janus kinases (JAKs) in response to stimulation by cytokines and chemokines. STAT3 has important functions in regulation of both innate and adaptive immunity and plays an important role in various autoimmune disorders including UC. Mudter et al. [21] reported an excessive accumulation of activated STAT3 in lamina propria monocytes of UC patients, and the elevated level of p-STAT3 was directly correlated with the degree of colonic tissue inflammation. MCP-1 is a downstream target of the STAT3 signaling pathway [7]. The results of our study demonstrated that Cit supplementation significantly inhibited the phosphorylation of STAT3 in colon tissue from UC rat group. This inhibition of STAT3 phosphorylation could lead to decreased MCP-1 expression, which in turn reduced monocyte infiltration in the inflamed colon tissue.

In conclusion, our study confirmed that Cit supplementation had a protective effect on TNBS-indiced colitis in rats. Cit supplementation prevented weight loss and structural impairement of the colon tissue integrity, reduced the number of peripheral blood monocytes, and inhibited the infiltration of monocytes in the colon tissue of UC rats. Cit supplementation reduced the expression of IL-6 and IL-17A in colon tissue of UC rats. In addition, Cit supplementation reduced the expression of MCP-1, which may be related to the inhibition of STAT3 phosphorylation. However, there are some limitations in our research. Since we underestimated the mortality caused by modeling, only 3 rats survived in the UC group, which may affect the reliability of our results to some extent. We suggest that Cit supplementation may be a potential treatment for UC; however, more in-depth studies are needed to confirm it. In addition, how Cit regulates the expression of MCP-1 and the phosphorylation of STAT3 also needs further study.

## Supporting information

S1 Fig Survival curves of each group. This study was carried out on 3 groups, each group contained 5 rats. After 7 consecutive days of intragastric administration, 5 rats in the NC group, 3 rats in the UC group and 4 rats in the UC+Cit group survived, respectively.
(TIF)

S1 Raw images (TIF)
