# Supplementary figures and images for: Protective effects of citrulline supplementation in ulcerative colitis rats
Source: PLoS One. 2020 Oct 16;15(10):e0240883. doi: 10.1371/journal.pone.0240883 (PMC7567373; doi:10.1371/journal.pone.0240883)

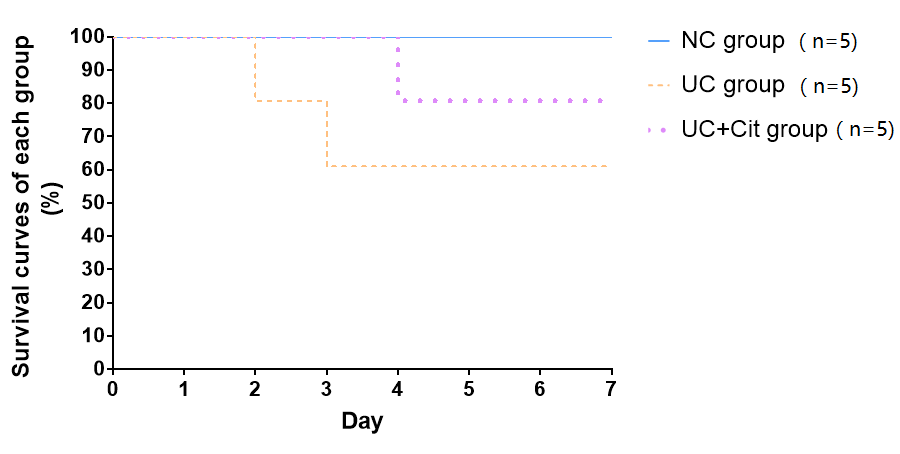

Supplement: S1 Fig — This study was carried out on 3 groups, each group contained 5 rats. After 7 consecutive days of intragastric administration, 5 rats in the NC group, 3 rats in the UC group and 4 rats in the UC+Cit group survived, respectively. (TIF) [file pone.0240883.s001.tif]

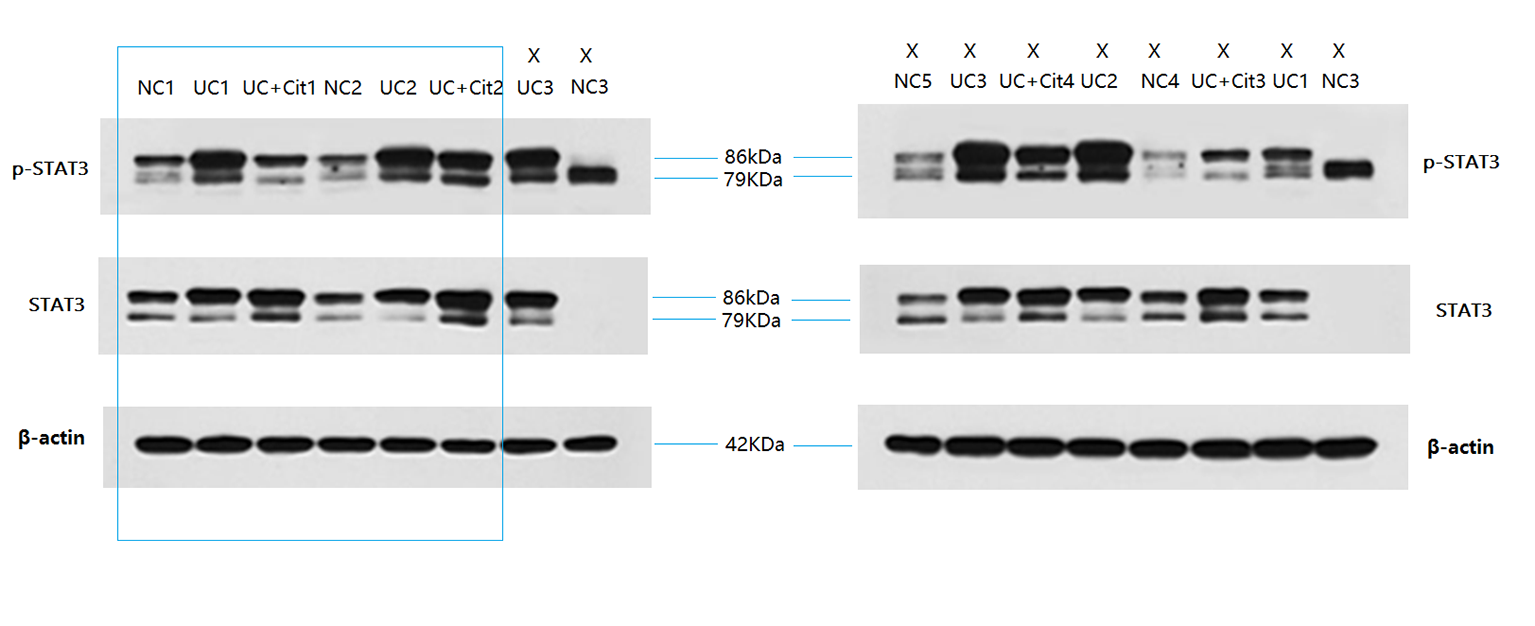

Supplement: S1 Raw images — (TIF) [file pone.0240883.s002.tif]
